# Thermotherapy Followed by Shoot Tip Cryotherapy Eradicates Latent Viruses and Apple Hammerhead Viroid from In Vitro Apple Rootstocks

**DOI:** 10.3390/plants11050582

**Published:** 2022-02-22

**Authors:** Jean Carlos Bettoni, Gennaro Fazio, Larissa Carvalho Costa, Oscar P. Hurtado-Gonzales, Maher Al Rwahnih, Abby Nedrow, Gayle M. Volk

**Affiliations:** 1 The New Zealand Institute for Plant and Food Research Limited, Batchelar Road, Palmerston North 4410, New Zealand; 2 USDA-ARS Plant Genetic Resources Unit, 630 W. North Street, Geneva, NY 14456, USA; Gennaro.Fazio@usda.gov (G.F.); akn6@cornell.edu (A.N.); 3 USDA-APHIS Plant Germplasm Quarantine Program, 9901 Powder Mill Road, Bldg 580, BARC-East, Beltsville, MD 20705, USA; larissa.carvalhocosta@usda.gov (L.C.C.); oscar.hurtado-gonzales@usda.gov (O.P.H.-G.); 4 Department of Plant Pathology, University of California-Davis, Davis, CA 95616, USA; malrwahnih@ucdavis.edu; 5 USDA-ARS National Laboratory for Genetic Resources Preservation, 1111 S. Mason Street, Fort Collins, CO 80521, USA; Gayle.Volk@usda.gov

**Keywords:** *Malus*, shoot tips, ASGV, ACLSV, AHVd, cryopreservation, droplet-vitrification

## Abstract

Virus and viroid-free apple rootstocks are necessary for large-scale nursery propagation of apple (*Malus domestica*) trees. Apple stem grooving virus (ASGV) and Apple chlorotic leaf spot virus (ACLSV) are among the most serious apple viruses that are prevalent in most apple growing regions. In addition to these viruses, a new infectious agent named Apple hammerhead viroid (AHVd) has been identified. We investigated whether thermotherapy or cryotherapy alone or a combination of both could effectively eradicate ACLSV, ASGV, and AHVd from in vitro cultures of four apple rootstocks developed in the Cornell-Geneva apple rootstock breeding program (CG 2034, CG 4213, CG 5257, and CG 6006). For thermotherapy treatments, in vitro plants were treated for four weeks at 36 °C (day) and 32 °C (night). Plant vitrification solution 2 (PVS2) and cryotherapy treatments included a shoot tip preculture in 2 M glycerol + 0.8 M sucrose for one day followed by exposure to PVS2 for 60 or 75 min at 22 °C, either without or with liquid nitrogen (LN, cryotherapy) exposure. Combinations of thermotherapy and PVS2/cryotherapy treatments were also performed. Following treatments, shoot tips were warmed, recovered on growth medium, transferred to the greenhouse, grown, placed in dormancy inducing conditions, and then grown again prior to sampling leaves for the presence of viruses and viroids. Overall, thermotherapy combined with cryotherapy treatment resulted in the highest percentage of virus- and viroid-free plants, suggesting great potential for producing virus- and viroid-free planting materials for the apple industry. Furthermore, it could also be a valuable tool to support the global exchange of apple germplasm.

## 1. Introduction

Apples (*Malus* spp.) are one of the most valuable fruit crops and are widely grown around the world [1]. Globally in 2019, the total apple area planted was 4.7 M hectares, producing about 87 Mt, which represents an increase in production of 35% despite a reduction of 16% of harvested area compared with 1999 [2]. This increase in productivity is primarily attributed to the use of higher density planting systems, made possible through the use of dwarfing rootstocks [3,4].

Vegetatively propagated apple rootstocks offer advantages over traditional seedling rootstocks because they consistently confer traits to scions that include dwarf growth habits, uniform plant architecture, improvements in fruit quality and nutrient assimilation, disease resistance, and tolerance to biotic and abiotic stress [5,6,7,8,9]. The Cornell–Geneva (Geneva^®^ series, CG) breeding program has developed several dwarfing rootstocks that are resistant to diseases and pests, and also provide precocious and consistent fruit yields [10,11,12,13,14]. However, the breeding of novel apple rootstocks from wild apple species has also introduced susceptibility to virus and virus-like diseases, with hypersensitive reactions observed within the Geneva rootstock series, G. 16, G. 814, and G. 935 [3,14,15,16,17]. There are variations in sensitivity to viruses and viroids in the Geneva series rootstocks, but the genetic basis of this resistance is unknown [5].

Virus and virus-like diseases have long been recognized as a major constraint for sustainable agricultural production on a global scale [18,19]. They are particularly problematic in vegetatively propagated crops, because new plants derived from infected sources continue to harbor the microbes, resulting in virus and viroid transmission from one generation to the next [20]. In addition to increased susceptibility to other pathogens, viral and viroid diseases can cause economic losses as a result of their negative effects on yield and fruit quality [21,22,23,24,25]. In commercially cultivated apple trees, the identification and characterization of the causal agents of virus-associated disease symptoms are challenging, as symptoms can often be confused with nutrient deficiencies, phytotoxicity, and the symptoms of other disease/plant stresses, furthermore, plants can sometimes be asymptomatic. Therefore, effective diagnostic tools are needed for early detection in propagation source materials to avoid the spread of diseased plants [19,26,27,28].

*Malus* is highly susceptible to virus/viroid infections. At least 21 viruses and 8 viroids are known to infect apple trees [20,21]. The latent viruses Apple chlorotic leaf spot virus (ACLSV, *Trichovirus*) and Apple stem grooving virus (ASGV, *Capillovirus*), species of the family *Betaflexiviridae*, are among the most damaging. Although ASGV and ACLSV infections are latent in most apple cultivars, symptoms including graft incompatibility, leaf and fruit malformation, stem grooving, chlorosis, and tree decline have been observed in sensitive cultivar–rootstock combinations [24,29,30]. As there are no known insect vectors, spread of ASGV and ACLSV is thought to occur through grafting of budwood and cuttings from infected plants [19]. Recently, a new infectious agent named Apple hammerhead viroid (AHVd, *Pelamoviroid;* [31]) has been identified in apple trees, associated with symptoms including trunk splitting, mosaic, necrosis, shoot decline, and dieback [32,33,34,35,36,37]. First identified in China in 2014 [32], AHVd is now globally widespread [32,33,34,35,36,37,38]. More research is needed to quantify the possible harmful effects of AHVd in apple trees [35,37,38,39,40]. 

Virus and viroid infections are typically managed in orchards by planting materials obtained from clean nursery stock. Once infected, there are no effective measures to remove viruses and viroids from orchard trees [18,41]. Improved methods for detection and clean-up of nursery stock materials are needed to ensure the availability of disease-free plants to the apple industry.

Shoot tip cryotherapy, liquid nitrogen (LN) treatment of in vitro plant shoot tips, can eliminate viruses from infected plants, including apple [42,43,44,45]. There is a range of efficacy reported in the literature that appears to be dependent upon the type of virus and apple cultivar [46,47]. Improvements in apple virus eradication, especially those that may infect the meristematic cells of the shoot tip, have been achieved by combining thermotherapy with cryotherapy [47,48]. However, to date, there are no reported studies on the use of cryotherapy or the combination of thermotherapy and cryotherapy to eradicate viroids from apple. We investigated whether cryotherapy and thermotherapy alone or in combination could effectively eradicate the latent viruses ACLSV and ASGV as well as AHVd from in vitro-cultured apple rootstocks.

## 2. Results

This research used in vitro plants of CG-series apple rootstocks that were infected with ACLSV (CG 2034), AHVd (CG 4213 and CG 5257), and ASGV (CG 6006), as initially determined by high throughput sequencing (HTS). HTS-based diagnostics of virus and viroids is a powerful tool that, combined with PCR-based pathogen detection procedures, rendered a reliable full-spectrum of virus/viroids agents present in the initial Geneva^®^ rootstocks used in this study. This information helped in determining (i) the extent of pathogen testing needed and (ii) the efficiency of each of the treatments presented here.

### 2.1. Survival and Regrowth of Shoot Tips 

For two replicates of each treatment, twenty shoot tips of each rootstock were excised, exposed to treatment conditions, and then assessed for survival and regrowth. Untreated shoot tip survival (100%) and regrowth (98%) levels were high for each of the four rootstocks (Table 1A,B). A four week thermotherapy (TT; 36 °C (day) and 32 °C (night)) resulted in a reduction in survival (86%) and regrowth (60%) compared with the shoot tip controls. Shoot tips that were treated with 2 M glycerol + 0.8 M sucrose for one day and then exposed to plant vitrification solution 2 (PVS2) for 60 or 75 min at 22 °C (no LN exposure) had survival levels of 83% and 75%, respectively, and regrowth levels of 48% and 39%, respectively. A combination of thermotherapy and PVS2 for 60 or 75 min reduced survival levels to 64% and 46%, respectively, and regrowth levels of 34% and 17%, respectively (Table 1A,B). 

Survival and regrowth levels were also assessed after LN exposures. Across the four cultivars, survival levels were 76% and 62% for PVS2 exposures of 60 or 75 min, followed by LN, compared with regrowth levels of 44% and 30% for the same treatments (Table 1A,B). Finally, thermotherapy combined with PVS2 exposures of 60 or 75 min had average survival levels of 52% and 31%, respectively, for the four rootstocks, and corresponding average regrowth levels of 15% and 4%. Thermo-treated shoot tips that were exposed to PVS2 for 75 min and LN treatment resulted in poor shoot tip regrowth ranging from 0% to 6% across the four rootstocks, as most of the surviving shoot tips produced a callus but failed to regenerate. While CG 4213 rootstock thermo-treated shoot tips that were exposed to PVS2 for 75 min and LN showed survival level of 30%, no plants were able to be regenerated.

### 2.2. Effect of Treatments on Virus and Viroid Eradication

After therapy treatments, in vitro recovered plants were transferred into pots in the greenhouse, grown on for four to five months, exposed to a dormancy period of four to six months, and then sampled after new leaves emerged once plants started growing again. A single greenhouse plant recovered from a shoot tip was pathogen-tested. Since the numbers of plants recovered varied among the therapy treatments, the number of pathogen-tested trees differed across the treatment conditions. The presence of ACLSV, ASGV, and AHVd was detected using reverse transcription polymerase chain reaction (RT-PCR). 

CG 2034 plants infected with ACLSV were identified in 69% (9 out of 13) of the plants derived from excised shoot tips, perhaps suggesting a patchy nature of ACLSV within the plants or some efficacy of virus eradication during the shoot tip excision process (Table 1C). Thermotherapy treatments, either with or without PVS2 exposure, and no LN resulted in a low percentages of CG 2034 plants free of ACLSV. PVS2 treatment for 60 min (no LN) resulted in 67% ACLSV-free CG 2034 plants. Liquid nitrogen increased the efficacy of ACLSV eradication, with 79% and 69% of the recovered plants free of ACLSV after PVS2 exposures for 60 or 75 minutes. In combination with thermotherapy, the eradication levels were 100% for both PVS2 60 and 75 min treatments, although the number of plants recovered diminished (Table 1A–C). 

All of the CG 6006 plants derived from shoot tips or after PVS2 exposures of 60 or 75 min (no LN) were infected with ASGV. Thermotherapy treatments decreased the infection level to 8% (TT), 11% (TT+PVS2 60 min), and 14% (TT + PVS2 75 min). Liquid nitrogen cryotherapy treatments (PVS2 exposures of 60 or 75 min) failed to eradicate ASGV; however, thermotherapy in combination with cryotherapy resulted in 100% eradication for 60 min PVS2 and 50% eradication for 75 min PVS2 exposures (Table 1C). 

CG 4213 and CG 5257 were infected with AHVd. The shoot tip, thermotherapy, PVS2, and thermotherapy + PVS2 treatments (no LN) were ineffective at removing AHVd from shoot tips of these two rootstocks. Similarly, cryotherapy with PVS2 treatments of 60 or 75 min also had no effect on eradication, as did thermotherapy + PVS2 for 75 min followed by LN. However, the four-week thermotherapy treatment of 36 °C (day) and 32 °C (night), in combination with PVS2 treatment for 60 min with LN, resulted in 25% (one out of four) AHVd-free plants of CG 4213 and 75% (six out of eight) AHVd-free plants of CG 5257. This combined treatment was the only effective treatment for AHVd elimination.

No visible morphological differences were apparent in the thermo- or/and cryo-treated plants after the initial four to five month growth period in the greenhouse. These plants were all screened for virus/viroid presence prior to dormancy. Nine CG 5257 plants did not exhibit AHVd after this first growth period in the greenhouse, but after the dormancy period, only six trees were free of AHVd. The other rootstock therapies had the same proportion of pathogen presence, before and after the dormancy period. Overall, approximately 70% of the recovered plants survived the greenhouse rooting and outdoor dormancy steps of this process.

**Table 1:** Shoot tips of *Malus* rootstocks infected with Apple chlorotic leaf spot virus (ACLSV), Apple hammerhead viroid (AHVd), or Apple stem grooving virus (ASGV) were excised from in vitro plants that were untreated (shoot tips), treated for four weeks at 36 °C (day) and 32 °C (night) (thermotherapy, TT), plant vitrification solution 2 (PVS2) for 60 or 75 min, or TT combined with PVS2 for 60 or 75 min without liquid nitrogen exposure (**A**). Shoot tips treated with PVS2 for 60 or 75 min or treated with TT followed by PVS2 for 60 or 75 min and LN (**B**). Quantities of pathogen-free plants identified after treatments (**C**).

**A**. Shoot tip survival after treatment (% ± SE)										
**Rootstock**	**No Liquid Nitrogen**						**Liquid Nitrogen**			
**Identity**	**Shoot Tips**	**Thermotherapy**	**PVS2 60 Min**	**PVS2 75 min**	**TT + PVS2 60 min**	**TT + PVS2 75 min**	**PVS2 60 min**	**PVS2 75 min**	**TT + PVS2 60 min**	**TT + PVS2 75 min**
CG 2034 (ACLSV)	100 ± 0 a	78 ± 3 abcdef	88 ± 3 abc	88 ± 3 abcd	68 ± 3 bcdefgh	50 ± 5 ghijkl	80 ± 5 abcde	73 ± 3 bcdefg	53 ± 3 fghijkl	30 ± 5 kl
CG 4213 (AHVd)	100 ± 0 a	88 ± 3 abc	88 ± 3 abc	78 ± 8 abcdef	55 ± 5 efghijk	38 ± 3 ijkl	80 ± 0 abcde	68 ± 3 bcdefgh	48 ± 3 ghijkl	30 ± 10 kl
CG 5257 (AHVd)	100 ± 0 a	90 ± 0 ab	93 ± 3 ab	80 ± 5 abcde	63 ± 3 cdefghi	53 ± 8 fghijkl	88 ± 3 abc	73 ± 3 bcdefg	55 ± 5 efghijk	28 ± 3 l
CG 6006 (ASGV)	100 ± 0 a	88 ± 3 abc	63 ± 8 cdefghi	60 ± 10 defghij	70 ± 5 bcdefg	42 ± 2 hijkl	55 ± 5 efghijk	35 ± 6 jkl	50 ± 7 ghijkl	34 ± 6 jkl
Mean	100 ± 0	86 ± 3	83 ± 7	75 ± 5	64 ± 3	46 ± 3	76 ± 7	62 ± 9	52 ± 2	31 ± 1
**B.** Shoot tip regrowth after treatment (% ± SE)										
**Rootstock**	**No Liquid Nitrogen**						**Liquid Nitrogen**			
**Identity**	**Shoot Tips**	**Thermotherapy**	**PVS2 60 min**	**PVS2 75 min**	**TT + PVS2 60 min**	**TT + PVS2 75 min**	**PVS2 60 min**	**PVS2 75 min**	**TT + PVS2 60 min**	**TT + PVS2 75 min**
CG 2034 (ACLSV)	100 ± 0 a	60 ± 10 cd	58 ± 8 cd	48 ± 3 cdefg	28 ± 3 efghijk	15 ± 5 hijk	43 ± 8 cdefgh	40 ± 5 cdefgh	15 ± 5 hijk	5 ± 5 jk
CG 4213 (AHVd)	95 ± 5 ab	55 ± 5 cde	43 ± 3 cdefgh	35 ± 10 defghi	25 ± 5 fghijk	15 ± 5 hijk	43 ± 3 cdefgh	28 ± 3 efghijk	10 ± 5 ijk	0 ± 0 k
CG 5257 (AHVd)	98 ± 3 a	58 ± 3 cd	48 ± 3 cdefg	43 ± 8 cdefgh	28 ± 3 efghijk	15 ± 0 hijk	50 ± 5 cdef	25 ± 5 fghijk	20 ± 5 ghijk	3 ± 3 k
CG 6006 (ASGV)	98 ± 3 a	68 ± 8 bc	43 ± 8 cdefgh	33 ± 3 defghij	55 ± 5 cde	24 ± 4 fghijk	40 ± 0 cdefgh	25 ± 5 fghijk	15 ± 3 hijk	6 ± 2 jk
Mean	98 ± 1	60 ± 3	48 ± 4	39 ± 3	34 ± 7	17 ± 2	44 ± 2	30 ± 4	15 ± 2	4 ± 1
**C.** Percent of non-infected plants obtained (number uninfected/total number assessed in parentheses) after treatment										
**Rootstock**	**No Liquid Nitrogen**						**Liquid Nitrogen**			
**Identity**	**Shoot Tips**	**Thermotherapy**	**PVS2 60 min**	**PVS2 75 min**	**TT + PVS2 60 min**	**TT + PVS2 75 min**	**PVS2 60 min**	**PVS2 75 min**	**TT + PVS2 60 min**	**TT + PVS2 75 min**
CG 2034 (ACLSV)	31 (4/13)	5 (1/19)	67 (8/12)	23 (3/13)	9 (1/11)	0 (0/6)	79 (11/14)	69 (11/19)	100 (6/6)	100 (2/2
CG 4213 (AHVd)	0 (0/6)	0 (0/20)	0 (0/2)	0 (0/4)	0 (0/6)	0 (0/6)	0 (0/7)	0 (0/9)	25 (1/4)	0 (0/0)
CG 5257 (AHVd)	0 (0/4)	0 (0/19)	0 (0/6)	0 (0/4)	0 (0/10)	0 (0/6)	0 (0/20)	0 (0/7)	75 (6/8)	0 (0/1)
CG 6006 (ASGV)	0 (0/5)	8 (1/12)	0 (0/5)	0 (0/7)	11 (1/9)	14 (1/7)	0 (0/10)	0 (0/3)	100 (6/6)	50 (1/2)

Values followed by different letters within each section were significantly different at *p* < 0.05 using Tukey’s mean separation test.

## 3. Discussion

Virus- and viroid-free plants are essential for sustainable breeding program activities, ensuring the global exchange of high health germplasm [49]. The development of efficient protocols for the production of virus- and viroid-free apple plants is fundamental for a successful apple industry. In the present study, the newly discovered AHVd viroid and the ASGV and ACLSV viruses were eradicated using a protocol that combined thermotherapy with cryotherapy. We found that a thermotherapy regime of alternating temperature at 36 °C (day) and 32 °C (night) for four weeks combined with cryotherapy, a PVS2 exposure duration of 60 min + LN, resulted in moderate to high efficacy of virus and viroid eradication in recovered plants. Shoot regrowth levels ranging from 10% to 20% were obtained across four CG rootstocks, with all tested plants of CG 6006 and CG 2034 found to be free of ASGV and ACLSV, while 75% and 25% of the CG 5257 and CG 4213 were found to be free of AHVd, respectively. Our results also demonstrated the importance of testing plant materials after a dormancy period to be certain that plants are free of viroids. Three plants that had negative test results for AHVd prior to dormancy were shown to have the viroid when regrown in outdoor potted plants following a dormant period. Although the efficacy of the combination of thermotherapy with cryotherapy has been reported for eradication of some apple viruses, to the best of our knowledge, this is the first study reporting success in eradicating of AHVd from infected in vitro-cultured apple rootstock plants.

Conventional methods such as meristem culture and thermotherapy followed by meristem culture are often used for virus eradication and have shown mixed results. In meristem culture, the size of the shoot tips is positively related to shoot regeneration, but negatively proportional to the virus eradication frequency. Li et al. [46] found that shoot regrowth levels of apple rootstocks ‘M9’ and ‘M26’ significantly increased, from 32.5% to 92.3%, as the size of shoot tips increased from 0.5 mm to 1.0 mm. While all the recovered plants from 0.5 mm shoot tips containing two leaf primordia were ASPV-free (Apple stem pitting virus), none of the recovered plants were free of ASGV. Wang et al. [50] also found that ASPV could be eradicated from 0.3 mm shoot tips containing two leaf primordia, but failed to remove ASGV from diseased in vitro-cultured ‘Gala’ apple shoots. The failure to eradicate ASGV is likely related to incipient infection in meristematic cells from apple shoot tips. ASPV has not been detected in the apical dome and first three leaf primordia, whilst ASGV was detected across the apical dome and leaf primordia of the shoot tips [46,50]. This information may explain why no ASGV-free plants were able to be produced in 1 mm shoot tips that were not thermo-treated in our study. The difficulty of excising such small meristems, combined with their low regeneration capacity and the inability to guarantee the removal of viral particles, are bottlenecks in meristem culture techniques [51,52].

Improved virus eradication is achieved when meristem excision is combined with thermotherapy [51]. Thermotherapy can reduce virus movement towards the meristematic cells by inhibiting viral replication and increasing RNA degradation. This decreases the viral particle load in infected shoot tips, permitting the use of larger meristems than those used for meristem culture without thermo-treatment [48,51,53,54,55]. Previous studies have shown that exposing plants to thermotherapy can be effective for ACLSV, ASGV, ApMV (Apple mosaic virus), and ASPV eradication in diseased apple cultivars [56,57]. The efficacy of producing virus-free apple plants varied according to the virus species and the nature of infection (single or mixed), cultivars, and the virus–host combination. Furthermore, the duration and temperature of the thermotherapy, as well as the size of the excised shoot tip affected the extent of virus eradication [48,51,58,59,60,61]. For example, Paprštein et al. [57] found that the success of thermotherapy in eradicating ACLSV, ASPV, and ASGV depended on the apple cultivar. In their study, shoot tips (1 to 2 mm) of apple cultivars ‘Idared’ and ‘Sampion’ were excised from shoots heat-treated at 39 °C for six and ten days. About 63% of ‘Idared’ and 44% of ‘Sampion’ shoot tips regenerated after the six-day thermotherapy period, and no shoot tips survived when duration of thermotherapy was extended to ten days. While four of five clones of ‘Idared’ were free of ACLV, ASPV, and ASGV, no virus-free plants were found in ‘Sampion’ [57]. Working on seven apple varieties, Hu et al. [56] also found that the effectiveness of thermotherapy in eradicating apple viruses was related to the cultivar and virus species. Incubation of pot-grown diseased plants at 38 °C for 30 days followed by excision of 1 mm shoot tips resulted in 71.4% (five out of seven plants) of the regenerated plants of ‘Apple 123’ being free of ACLSV and ASPV, and one out of two regenerated plants of ‘Huafu’ being free of ACLSV, ASGV, ASPV, and ApMV. None of the recovered plants from other varieties were free of virus [56]. In the present study, an alternating temperature of 36 °C (day) and 32 °C (night) for four weeks followed by the excision of 1 mm shoot tips resulted in low efficiency of ASGV (8%) and ACLSV (5%) eradication, and did not produce any AHVd-free plants. Zhao et al. [48], using the same thermotherapy condition as the present study, found frequencies of ASGV eradication of 20% and 41% in shoot tips (1.5 mm) excised from thermo-treated in vitro shoots of ‘Gala’ for four and six weeks, respectively. In general, prolonged heat treatment associated with high temperatures can increase the frequency of virus eradication, but concurrently reduces the viability of the treated explants, and often the host plants are sensitive to these conditions [51,57,59,62,63].

In recent years, studies have demonstrated efficient eradication of viruses using shoot tip cryotherapy [59,64,65,66,67,68,69,70,71,72]. Cryotherapy makes use of LN exposure (−196 °C) to selectively kill vacuolated and differentiated cells that harbor viruses within shoot tips [64,65,73]. Although cryotherapy is based on cryopreservation protocols, unlike cryopreservation, the goal of this technique is to induce significant damage to virus-infected tissues surrounding the meristem to maximize the chances of obtaining virus-free plants after cryotherapy, rather than preservation of tissue per se [74]. 

To date, four apple viruses have been eradicated by shoot tip cryotherapy: ACLSV, ASPV, ASGV, and ApMV [42,43,44,45,46,47]. As with the other virus eradication techniques mentioned above, the eradication efficiency is affected by specific interactions between pathogen and apple genotype as well as virus species. Although cryotherapy can eradicate viruses from apple tissues, some of the recovered plants were still pathogen-infected [42,43,44,45,46,47]. In all studies using cryotherapy, ASGV has proved more difficult to eradicate than other virus species, regardless of the cryotherapy technique. We (Bettoni et al. [43,44] and Souza et al. [45]) and Romadanova et al. [42] previously reported that ASGV could be eradicated by cryotherapy at a lower frequency than ACLSV, ASPV, and ApMV in ‘Marubakaido’ apple rootstock (*Malus prunifolia*) and ‘Sinap Almatinskyi’ (*Malus* × *domestica*). Similarly, Liu et al. [47] found that ASGV eradication efficacy was apple cultivar dependent. Two out of sixteen ‘Rui Xianghong’ (‘Fuji’ × ‘Cripp’s Pink’) plants were ASGV-free, and all of the tested ‘Qinyue’ (‘Fuji’ × ‘Gala’) plants were still ASGV infected after cryotherapy. Li et al. [46] and Zhao et al. [48] found that cryotherapy failed to eradicate ASGV from diseased in vitro-cultured shoots of apple rootstocks ‘M9’ and ‘M26’ and ‘Gala’. Our current results with Geneva^®^ apple rootstock CG 6006 (PK-14 x ‘Robusta 5’) were consistent with those reported by Li et al. [46] and Zhao et al. [48], where none of the plants were ASGV-free after cryotherapy. Using the same cryotherapy protocol, ACLSV eradication from CG 2034 cryo-treated shoot tips was up to 69%, but there was no AHVd eradication in CG 5257 and CG 4213. These results demonstrate that, like ASGV, the AHVd viroid is also difficult to eradicate. It is well-known that some viroids can infect meristematic cells of shoot tips [75,76,77]. AHVd cellular localization studies have not been performed, but it may be present in meristematic cells, which could contribute to the challenge of eradication. 

Improved virus eradication can be achieved by combining thermotherapy with cryotherapy, rather than using each technique alone [47,48,51,70,78,79,80]. In combination, thermotherapy and cryotherapy work synergistically by inhibiting virus movement to produce larger virus-free areas in the apical meristem, inducing enlargement of vacuoles in shoot tips’ cells, and therefore making infected cells more susceptible to cryo-injury in infected differentiated cells [53]. While shoot tip cryotherapy alone failed to eradicate ASGV from in vitro diseased ‘Gala’ apple shoots, the combination of thermotherapy followed by cryotherapy resulted in 100% ASGV-free plants. Similarly, Wang et al. [53] and Mathew et al. [79] found that shoot tip cryotherapy or thermotherapy alone did not eradicate Raspberry bushy dwarf virus (RBDV) from in vitro infected raspberry (*Rubus idaeus*); however, a combination of thermotherapy and cryotherapy resulted in 33% to 35% and 48.6% RBDV-free plants, respectively. In the present study, a combination of thermotherapy and cryotherapy successfully eradicated ASGV and ACLSV as well as AHVd viroid from diseased in vitro-cultured apple rootstocks. Although a combination of thermotherapy (four weeks at 36 °C (day) and 32 °C (night)) and cryotherapy (PVS2 exposure duration of 60 min + LN) led to a decrease in shoot regrowth, all tested plants were free of ASGV (CG 6006) and ACLSV (CG 2034), whilst 25% (CG 4213) to 75% (CG 5257) were free of AHVd. This procedure therefore appears to have great potential to assist in the production and supply of virus- and viroid-free planting materials for the apple industry, as well as accelerating current therapy conditions used by quarantine programs. Furthermore, it might also be a valuable tool to support the global exchange of germplasm in association with breeding program activities.

The current Animal and Plant Health Inspection Service, US Department of Agriculture (USDA-APHIS) Plant Germplasm Quarantine Program’s (PGQP) thermotherapy conditions of 38 °C day, 32 °C night for 9 to 11 weeks followed by the excision of 0.2 to 0.5 mm shoot tips are 70% to 95% effective for a wide range of viruses that are intercepted in imported apple germplasm. These viruses include ASPV, ASGV, ACLSV, Apple green crinkle associated virus (AGCaV), Citrus concave gum-associated virus (CCGaV), and Apple rubbery wood-associated viruses 1 and 2 (ARWaV-1, ARWaV-2) (Hurtado-Gonzales, personal communication). The methodologies reported in the current study represent an effective reduction in time (five to seven weeks shorter) and skills needed (as shoot tips excised can be larger), that together could streamline the process of virus or viroid elimination, which is the major bottleneck for quarantine programs that must deliver virus/viroid tested germplasm. 

In addition, apple scion breeding programs often rely on grafting new germplasm onto rootstocks (that may be virus infected) to evaluate, distribute, and commercialize material. Similarly, apple rootstocks are evaluated by grafting various scions that may or may not be free of viral particles. These practices sometimes cause the scions or rootstocks to be infected with pathogens. As a result, it is necessary to test and eradicate pathogens as needed in apple scion and rootstock released prior to commercialization. The techniques described herein will contribute to development and maintenance of healthy apple nursery stock. 

## 4. Materials and Methods 

### 4.1. Plant Materials and Growth Conditions 

**Figure 1:**
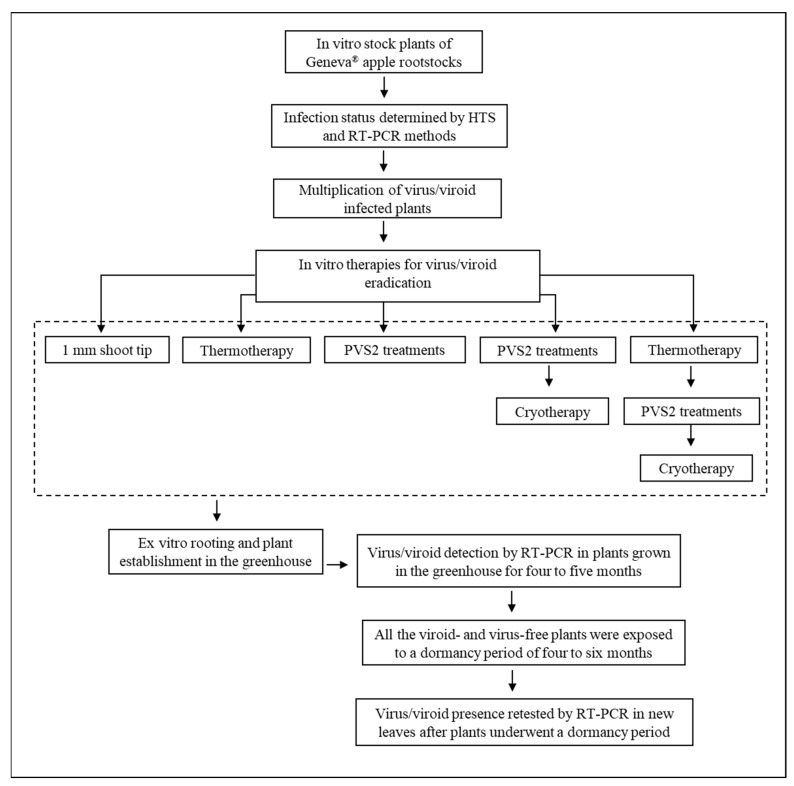
Flowchart depicting the workflow of this study. In vitro therapies were used for eradication of viroid and viruses from infected in vitro-grow Geneva^®^ apple rootstock shoots. Following treatments, shoot tips were recovered on growth medium, transferred to the greenhouse, and their sanitary status was checked by RT-PCR in plants grown in the greenhouse for four to five months. Viroid and virus-free plants were exposed to a dormancy period of four to six months, and then grown again prior to sampling leaves to retest for the presence of viruses and viroids.

Tissue cultures of Geneva^®^ apple rootstocks CG 4213 and CG 5257 (both ‘Ottawa 3’ × ‘Robusta 5’), CG 6006 (PK-14 × ‘Robusta 5’), and CG 2034 (‘Dolgo crab’ × ‘Malling 27’) were originally sent from the Agricultural Research Service, US Department of Agriculture (USDA-ARS) Plant Genetic Resources Unit (PGRU), Geneva, NY, USA to the USDA-ARS National Laboratory for Genetic Resources Preservation in Fort Collins, CO, USA. Infection status of in vitro stock plants was determined by HTS and RT-PCR methods (see below). Apple rootstock CG 2034 was infected with ACLSV, CG 6006 with ASGV, and CG 5257 and CG 4213 with AHVd. In vitro plants were maintained and subcultured every four weeks in Magenta^®^ GA7 culture vessels (77 × 99 mm) with 80 mL of shoot multiplication medium (SMM) composed of Murashige and Skoog [81] medium (MS) (PhytoTechnology Laboratories®, Lenexa, KN, USA containing 30 g L^−1^ sucrose, 0.1 g L^−1^ myo-inositol, 1 mg L^−1^ 6-benzyl aminopurine (BA), 0.3 mg L^−1^ indole−3-butyric acid (IBA), 0.2 mg L^−1^ gibberellic acid (GA3), and 7.5 g L^−1^ agar at pH 5.5 (pH 5.8 prior to autoclaving) at a density of five shoots per vessel. Cultures were grown at 25 °C under a photoperiod of 16 h light day with a light intensity of 40 μM m^−2^ s^−1^. Figure 1 shows the workflow of this study.

### 4.2. In Vitro Therapies for Virus/Viroid Eradication 

#### 4.2.1. Thermotherapy Treatments

**Figure 2:**
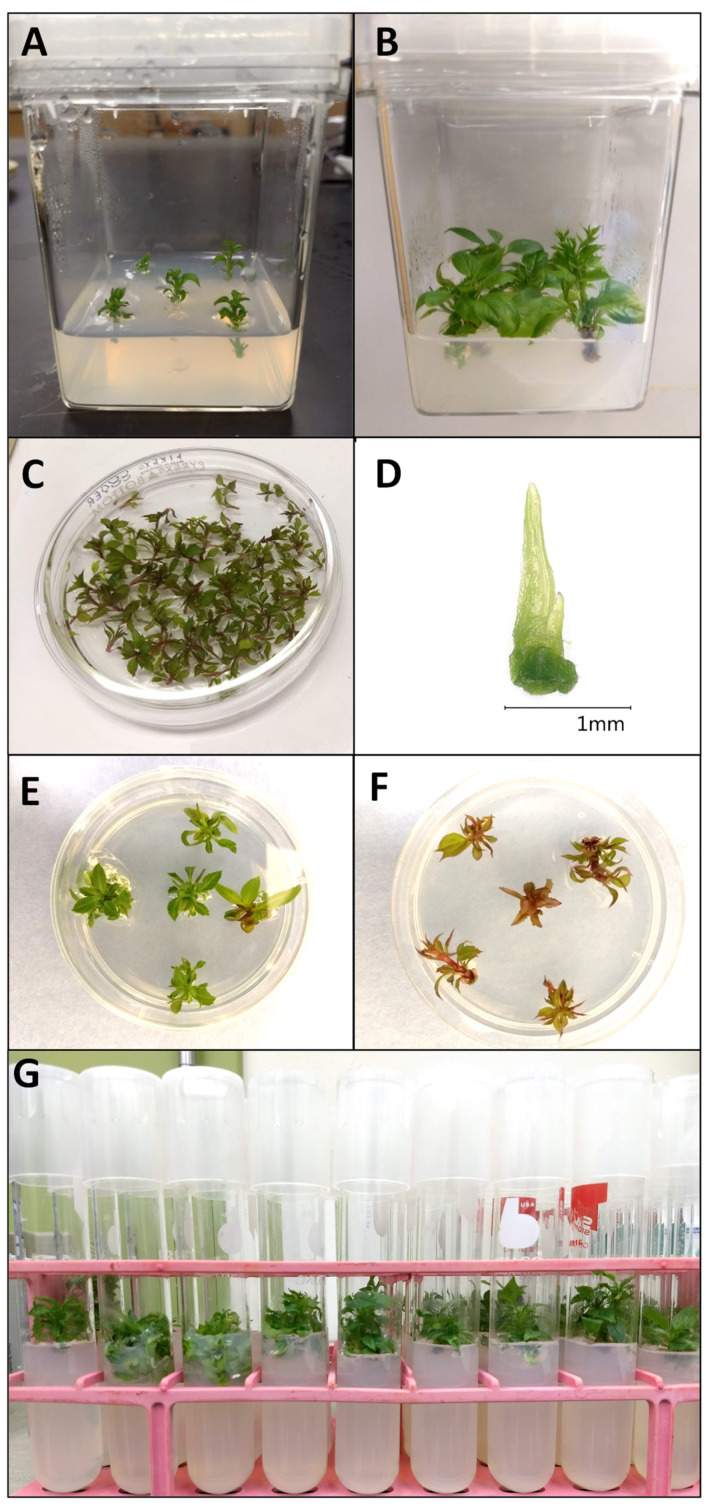
In vitro growing *Malus* plants undergoing virus eradication therapy treatments, (**A**) CG 5257 prior to thermotherapy exposure, (**B**) CG 5257 after 4 weeks of thermotherapy, (**C**) CG 6006 prior to shoot tip excision, (**D**) 1 mm shoot tip excised from CG 5257, (**E**) CG 5257 exhibiting regrowth after eight weeks, (**F**) CG 6006 exhibiting regrowth after eight weeks, and (**G**) treated in vitro plants prior to shipment to Geneva, New York for greenhouse plant establishment.

Thermotherapy procedures were performed as described by Zhao et al. [48], with the following modifications. Shoot segments (1.5 cm in length) containing the apical bud and two to three axillary buds were excised from four-week-old cultures and placed in Magenta^®^ GA7 culture vessels (77 × 99 mm) with 80 mL SMM at a density of five shoots per vessel and cultured at the same conditions as stock cultures (Figure 2A). After two weeks of culture, vessels containing the shoots were moved into a growth chamber (E-36VL; Percival^®^ Scientific, IA, USA) set at alternating temperatures of 36 °C (day) and 32 °C (night) under a 16-h photoperiod with a light intensity of 90 μM m^−2^ s^−1^. Apical shoot tips 1 mm in length and containing two to three leaf primordia were excised from heat-treated in vitro diseased shoots after four weeks of thermotherapy (Figure 2B,C).

#### 4.2.2. PVS2 and Droplet-Vitrification Cryotherapy Treatments

Apical shoot tips of 1 mm length containing two to three leaf primordia (Figure 2D) were excised from four-week-old shoots and subjected to droplet-vitrification cryotherapy, as described by Li et al. [82], with modifications. Excised shoot tips were inoculated on basal medium (BM; MS containing 30 g L^−1^ sucrose, 0.25 mg L^−1^ BA, 0.01 mg L^−1^ IBA, and 2.6 g L^−1^ of gellan gum at pH 5.5 (pH 5.8 prior to autoclaving)) overnight at 25 °C in the dark. Shoot tips were then incubated on preculture medium (MS with 2M glycerol, 0.8M sucrose, and 7.5g L^−1^ of agar at pH 5.8 (pH 6.4 prior to autoclaving)) for one day at 25 °C in the dark, followed by exposure to PVS2 (filter-sterilized half-strength MS with 0.4 M sucrose, 30% (*w*/*v*) glycerol, 15% (*w*/*v*) ethylene glycol, and 15% (*w*/*v*) dimethyl sulfoxide (DMSO) at pH 5.8, [83]) at 22 °C for 60 or 75 min. For LN exposures, 2 min before the end of each treatment, PVS2-treated shoot tips were placed onto a thin layer of PVS2 on sterile aluminum foil strips (~6 × 25 mm) and then plunged into LN. After one hour of LN exposure, the aluminum foil strips with shoot tips were warmed quickly by inverting the strips in unloading solution (half-strength MS with 1.2 M sucrose at pH 5.7 (pH 7.5 before autoclaving)) at 22 °C and for 20 min.

#### 4.2.3. Thermotherapy and Droplet-Vitrification Cryotherapy

Apical shoot tips of 1 mm in length containing two to three leaf primordia (Figure 2D) were excised from in vitro shoots after four weeks of thermotherapy and subjected to PVS2 exposure and/or droplet-vitrification cryotherapy, as described in Section 4.2.2.

### 4.3. Shoot Tip Recovery and Data Analyses

Shoot tips that underwent the in vitro therapies were placed onto BM overnight in the dark at 25 °C, then transferred to fresh BM and cultured for two weeks in darkness. Finally, they were grown in the light at 25 °C (40 μM m^−2^ s^−1^, 16 h photoperiod). Regenerated shoots with approx. 1 to 2 cm of growth were transferred to individual test tubes containing BM medium to avoid possible cross-contamination (Figure 2G). 

Shoot tip survival (shoot tips that exhibited green cell mass growth or leaf tissue) and regrowth (shoot tips exhibiting organized shoots with a new leaf emerging) were measured eight weeks after placing shoot tips on recovery media (BM) (Figure 2E,F). Each experiment was performed with at least two replicates of 20 shoot tips for each treatment. Means and standard errors were calculated across experimental replicates and the least significant differences were analyzed using one-directional ANOVA and Tukey’s test at *p* ≤ 0.05. 

### 4.4. Ex Vitro Rooting, Acclimatization and Plant Maintenance

Plants recovered from treated shoot tips for three months with up to twenty plantlets were randomly selected for shipment to Geneva, NY for greenhouse establishment. Regenerated shoots were multiplied using the same conditions as stock cultures on multiplication medium (MS containing 30 g L^−1^ sucrose, 1 mg L^−1^ IBA, 1 mg L^−1^ BAP, 0.2 mg L^−1^ GA3, 0.1 g L^−1^ myo-inositol, and 8 g L^−1^ agar at pH 5.8) in Magenta^®^ GA7 culture vessels (77 × 99 mm) to provide enough shoots to be rooted. Shoots were then rooted using liquid induction followed by ex vitro rooting. Three weeks after the last subculture, a small amount (approximately 5 mL) of the liquid induction solution (MS containing 30 g L^−1^ sucrose, 1 mg L^−1^ IBA, 0.2 mg L^−1^ GA3 acid, and 0.1 g L^−1^ myo-inositol at pH 5.8) was poured into the culture vessel until the base of the plantlets was submerged. After two more weeks of culture, microcuttings were harvested and pruned to 2 cm in length, with all leaves removed except the top two to four leaves. The stems of the microcuttings were then submerged up to the leaves in sterilized potting soil mix (Lambert^®^ LM-3, Rivière-Ouelle, QC, Canada) contained in a clam-shell deli container (Genpak^®^, Greens Falls, NY, USA). Each container received a fine mist of distilled water over the plants and the lid was completely closed to maintain high relative humidity. The plants were maintained in a growth room with a 16 h photoperiod under four standard fluorescent tube lamps measuring approximately 300 lux of photosynthetically active radiation and at 25 °C, with the bottom of the containers heated at 22 to 24 °C. After six weeks, the lid was gradually removed to acclimate plants. When plants had grown at least four new leaves, they were transplanted into Deepots^®^ (Stuewe & Sons Inc.,Tangent, OR, USA) (656 mL capacity) plastic pots containing potting soil mix (Lambert^®^ LM-3, Rivière-Ouelle, QC, Canada) and moved to the greenhouse. The only special care given was careful transplanting and gentle watering for the first week. They were grown in a greenhouse at 24 °C (day) and 21 °C (night) under a 14 h photoperiod. Plants were watered regularly and fertilized with commercial fertilizer (Jack’s Professional^®^ water-soluble acid fertilizer 21-7-7; J R Peters Inc, Allentown, PA, USA), and integrated pest management was used to control diseases and pests.

A maximum of one plant established in soil from a recovered shoot tip was used for virus/viroid testing by RT-PCR; therefore, the number of plantlets used for diagnosis varied depending on the available samples obtained in each treatment.

### 4.5. Virus/Viroid Detection

The sanitary status of the in vitro stock plants used for virus eradication therapies was determined using HTS. HTS-based diagnosis of stock plants revealed the presence of single virus/viroid infection of ACLSV for line CG 2034, ASGV for line CG 6006, and AHVd in lines CG 4213 and CG 5257. These results were also confirmed by RT-PCR. The presence of viruses and viroids in apple rootstocks that underwent the in vitro therapies and controls was assessed at two different stages using RT-PCR, as recommended by molecular testing guidelines to process apple quarantine material (APHIS Plant Protection and Quarantine, Plants for Planting, 2021). The first test was undertaken after plants were initially established in the greenhouse for four to five months and then all samples from each of the corresponding treatments that initially resulted negative were retested (second test) after a dormancy period of four to six months in plants outside in pots.

#### 4.5.1. HTS-Based Diagnostic in Stock Plants

A subgroup of RNA extracts from apple samples from each of the rootstock lines was subjected to HTS for virus/viroid detection. Briefly, total RNA from young leaves was extracted using the GenCatch TM Plant RNA Purification Kit (Epoch life science, Missouri City, TX, USA) combined with the RLT lysis buffer (Qiagen, Germantown, MD). RNA concentration of each sample was measured using Qubit (Qiagen), and RNA integrity number (RIN) was determined using the Agilent 4200 TapeStation system (Santa Clara, CA, USA). Libraries for high quality RNA samples (RIN > 6) were then prepared using the Ribo-Zero™ Plant Leaf Kit for ribodepletion followed by TruSeq Stranded Total RNA Library Prep Kit (Illumina, San Diego, CA, USA), according to the manufacturer’s instructions. After quantification and normalization, libraries were sequenced in a 75-cycle high output flow cell on the Illumina NextSeq 500 platform in the APHIS-PGQP sequencing laboratories (Beltsville, MD, USA).

HTS data analysis was performed as described by Malapi-Wight et al. [84]. Briefly, after de-multiplexing and adapter trimming using bcl2fastq (v.2.20.0.422) Conversion Software 1.8.4. (Illumina), the quality of the sequences was analyzed by FASTQC [85]. Raw reads were filtered and trimmed using Trimmomatic (v.0.39) [86] with parameters “Leading:3, Trailing:3, SlidingWindow:4:20, Minlen:36”. Trimmed reads were assembled using the de novo assembly tool SPAdes (v3.13.0) [87]. The generated contigs were then compared with the NCBI viral database and Reference Viral Database (RVDB) [88] using a 10^−3^ e-value cut-off. Top hit contigs were inspected and BLASTN was used to confirm the virus identity.

#### 4.5.2. Virus/Viroid Diagnostics Using RT-PCR

Total RNA extracts from leaves of all plants regenerated after the in vitro therapies and controls were screened by RT-PCR for the presence of the virus/viroid previously detected in untreated cultures, such as ACLSV, ASGV, or AHVd. The sequences of the three primer pairs used are listed in Table 2. Primers targeting NAD5 (NADH-ubiquinone oxidoreductase chain 5) gene from the host plant were also included in all PCR reactions. RT-PCR for ACLSV was carried out using a Platinum PCR SuperMix High Fidelity kit (Invitrogen, Carlsbad, CA, USA) with 150 nM of the forward and reverse primers for the virus, 50 nM of the forward and reverse primers for NAD5, and 1 µl of RNA template in a final reaction volume of 25 µL. The PCR program consisted of an initial cDNA step for 30 min at 50 °C followed by 15 min at 95 °C, and 40 cycles of 30 s at 94 °C, 45 s at 55 °C, and 2 min at 72 °C, and one final extension of 10 min at 72 °C. For ASGV, the RT-PCR reactions were performed using a One-Step RT-PCR Kit (Qiagen) with 600 nM of the forward and reverse primers for the virus, 200 nM of the forward and reverse primers for NAD5, and 1 µL of RNA template in a final reaction volume of 25 µL. The following cycling conditions were used: 50 °C for 45 min, 95 °C for 5 min, followed by 35 cycles at 94 °C for 1 min, 58 °C for 1 min, and 72 °C for 2 min, and one final extension at 72 °C for 10 min. For AHVd, the SuperScript III Platinum One-Step RT-PCR kit (Invitrogen) was used, with 250 nM of the forward and reverse primers, 62 nM of the forward and reverse primers for NAD5, and 1 µL of RNA template in a final reaction volume of 20 µL. The PCR program used for amplification was as follows: 50 °C for 30 min, 94 °C for 2 min, followed by 35 cycles at 94 °C for 30 s, 59 °C for 1 min, and 68 °C for 40 s, and one final extension at 68 °C for 5 min. The amplified fragments were visualized using the QIAxel capillary gel electrophoresis system (Qiagen, Irvine, CA, USA).

## Figures and Tables

**Table 2:** Primers and probes used for detection of ACLSV, ASGV, AHVd, and NAD5 in apple rootstock samples.

Primer	Sequence	Product Size	Source
ACLSV-F	5′-TTCATGGAAAGACAGGGGCAA-3′	677 bp	[89]
ACLSV-R	5′-AAGTCTACAGGCTATTTATTATAAGTCTAA-3′		
ASGV-F ASGV-R	5′-GCCACTTCTAGGCAGAACTCTTTGAA-3′	273 bp	[89]
	5′-AACCCCTTTTTGTCCTTCAGTACGAA-3′		
AHVd-F AHVd-R	5′-CCCTCCGGTCKTRTCCAACC-3′	92 bp	Costa et al., unpublished
	5′-GCGAGAGAGAGCGACTTCTC-3′		
NAD5-F NAD5-R	5′-GATGCTTCTTGGGGCTTCTTGTT-3′	181 bp	[89]
	5′-CTCCAGTCACCAACATTGGCATAA-3′		

Abbreviations: ACLSV Apple chlorotic leaf spot virus, ASGV Apple stem grooving virus, AHVd Apple hammerhead viroid, NAD5 NADH-ubiquinone oxidoreductase chain 5, bp base pairs.

## Data Availability

The data presented in this study are available on request from the corresponding author.

## Abbreviations

ACLSV	Apple chlorotic leaf spot virus
AGCaV	Apple green crinkle associated virus
AHVd	Apple hammerhead viroid
ApMV	Apple mosaic virus
ARWaV-1	Apple rubbery wood-associated viruses 1
ARWaV-2	Apple rubbery wood-associated viruses 2
ASGV	Apple stem grooving virus
ASPV	Apple stem pitting virus
BA	6-benzyl aminopurine
BM	Basal medium
CCGaV	Citrus concave gum-associated virus
DMSO	Dimethyl sulfoxide
GA3	Gibberellic acid
HTS	High-throughput sequencing
IBA	Indole-3-butyric acid
LN	Liquid nitrogen
MS	Murashige and Skoog medium
NAD5	NADH-ubiquinone oxidoreductase chain 5
PVS2	Plant vitrification solution 2
RBDV	Raspberry bushy dwarf virus
RT-PCR	Reverse transcription polymerase chain reaction
RVDB	Reference viral database
SMM	Shoot multiplication medium
